# The “B.U.M.P.” Concept: Combined Prosthetic and Surgical Approach in Esthetic Immediate Implant Placement. A Case Report

**DOI:** 10.3390/healthcare14142107

**Published:** 2026-07-14

**Authors:** Giacomo Dallari, Valentina Bentivogli, Jonathan Esquivel, Matteo Sangiorgi, Ilham Mounssif, Lorenzo Breschi, Giovanni Zucchelli, Martina Stefanini

**Affiliations:** 1 Department of Biomedical and Neuromotor Sciences, University of Bologna, 40125 Bologna, Italy; dallari@mail.com (G.D.); matteo.sangiorgi7@unibo.it (M.S.); ilham.mounssif2@unibo.it (I.M.); lorenzo.breschi@unibo.it (L.B.); martina.stefanini2@unibo.it (M.S.); 2 Independent Researcher, Metairie, LA 70005, USA; jesquiveldds@gmail.com; 3 Department of Periodontology, Dental School of Dental Medicine, University of Bern, 3012 Bern, Switzerland; zucchelli@giovannizucchelli.eu

**Keywords:** immediate implant placement, interdental papilla, peri-implant soft tissue augmentation, esthetic zone, connective tissue graft, prosthetic restoration, soft tissue management

## Abstract

**Background/Objectives**: Immediate implant placement in the esthetic zone is increasingly preferred due to its advantages for patient comfort and reduced treatment time. However, maintaining peri-implant soft tissue volume and preserving or reconstructing the interdental papilla remain significant clinical challenges. This report aims to describe a clinical protocol designed to enhance peri-implant soft tissue stability and promote papilla regeneration following immediate implant placement. **Methods**: This article presents a clinical case managed through a combined mucogingival and prosthetic approach. The protocol included digital planning for implant positioning, immediate implant placement, and a connective tissue graft to support peri-implant soft tissues. Particular attention was given to the design and progressive modification of the provisional restoration. Targeted additions of composite material were applied in the under-proximal areas of the provisional crown to create controlled pressure at the base of the papilla, aiming to stimulate soft tissue adaptation and growth. **Results**: The progressive “bumping” technique applied to the provisional crown promoted the gradual growth of the interdental papilla and complete closure of the interproximal spaces. Clinical and radiographic follow-up showed stable peri-implant soft tissues, adequate tissue volume, and highly satisfactory esthetic outcomes. **Conclusions**: This report represents an initial proof of concept that highlights the importance of integrating precise surgical techniques, customized prosthetic contouring, and digital workflows to achieve predictable soft tissue management in immediate implant placement. This combined approach may contribute to improved papilla formation and enhanced esthetic outcomes. Further prospective studies are needed to confirm the reproducibility and clinical advantages of this protocol.

## 1. Introduction

In the context of tooth replacement in the esthetic area, immediate implant placement (IIP) and provisionalization have become the treatment of first choice, due not only to enhanced patient satisfaction (fewer surgical steps, lower morbidity, no need for a removable provisional prosthetic), but also because the implant survival rate is comparable to that of early and delayed implant placement [1].

Unfortunately, IIP is unable to mitigate buccal hard- and soft-tissue remodeling following tooth extraction [2,3]. Inadequate management of the preservation or reconstruction of hard and soft peri-implant tissues can, over time, lead to an impaired esthetic outcome, such as soft tissue dehiscence and/or incomplete papilla fill. The shape of the interdental papilla is influenced by the contact points between adjacent teeth, the width of the interproximal tooth surfaces, and the cemento-enamel junction (CEJ). The interdental papilla is pyramidal in shape at the anterior teeth, while in posterior regions there are two papillae joined by a concave saddle region called a ‘col’. The concomitant loss of attachment in the adjacent teeth can result in greater difficulty in obtaining an adequate reconstruction of the interproximal papilla [4]. For this reason, it is very important to avoid bone loss at neighboring sites when performing an immediate post-extraction implant.

To reduce hard and soft tissue dimensional changes after tooth extractions, various procedures have been suggested: grafting into the implant-socket bone wall gap [5], connective tissue graft (CTG) [6], papilla reconstruction [7], hyaluronic acid or autologous fibroblasts injections [8,9], and guided tissue regeneration [10]. In addition to surgical techniques, the correct prosthetic approach seems to be fundamental in contouring and supporting soft tissues, as well as promoting papilla formation [11].

Among the key determinants of esthetic success in implant dentistry, peri-implant papilla formation remains one of the most challenging to achieve. Limited vascularization and the absence of a periodontal ligament often constrain soft tissue regeneration.

A large number of techniques exist for peri-implant papilla reconstruction, with multiple surgical approaches and, in some cases, multiple surgical interventions necessary to achieve satisfactory results, before, during, and after implant placement. The “Iceberg” Connective Tissue Graft (iCTG) technique [12] combines interproximal tissue reconstruction with a composite graft obtained by splitting a second connective tissue graft from the maxillary tuberosity into two pieces and suturing them to the ends of a wider palatal graft. This composite graft is then sutured on top of the implant platform, using the periosteum and the papillae of the adjacent teeth as anchorage points, thereby promoting primary closure and vertical soft-tissue augmentation. This configuration enhances soft tissue thickness and compensates for deficient interproximal bone peaks, improving papilla height and contour. A further soft tissue increase can be obtained with a second surgical procedure called the “Garage approach” [13]: a strip CTG combined with a CTG inserted underneath a pouch prepared into the previous iCTG at the level of the crest can obtain an increase in the soft tissue volume, which can be advocated especially in an aesthetic area.

The “trouser technique” proposed by Pavon et al. [14] is another novel connective tissue graft approach aimed at simultaneous buccal and interproximal soft tissue augmentation in aesthetic areas. A trouser-shaped CTG, fixed under a tunnelled flap, demonstrated a significant volumetric gain in the coronal and interproximal areas with limited patient morbidity.

The “distally anchored connective tissue graft platform” [15] approach involves folding an autogenous connective tissue graft and anchoring it distally to improve soft-tissue volume in the papillary region. Clinically, the method seemed to yield stable mucosal margins and complete papilla fill, with high patient satisfaction.

In addition to surgical techniques, the correct prosthetic approach is fundamental for contouring and supporting soft tissues and for promoting papilla formation [11]. Furthermore, the concomitant presence of attachment loss in the adjacent teeth can result in greater difficulty in obtaining an adequate reconstruction of the interproximal papilla [4].

The correct three-dimensional position of the implant and its design (bone-level or tissue-level, platform switching) can influence the shape of the emergence profile [16]. A harmonic contour with respect to the gingival tissues of the provisional restoration is essential to achieve a proper final esthetic rehabilitation [17,18,19].

It has been stated that IIP Type 1 is the treatment of choice with a flapless approach in specific anatomic conditions, such as intact facial bone walls with a thick-wall phenotype (>1 mm) and a thick gingival biotype [20]. According to the literature, only 5–10% of single-tooth extraction sites could fulfill these criteria [21]. Moreover, flapless post-extraction implant placement has been associated with a series of esthetic and functional complications due to impaired visibility; this leads to an incorrect implant position/angulation with the anatomical level of the buccal bone crest and the adjacent teeth or implants. Consequently, mucosal recessions, bone fenestrations, dehiscence, or a combination of the last two may occur, especially during freehand implant placement [22,23,24,25].

Computer-guided surgery can allow predictable implant positioning and good prosthetic finalization even in flapless approaches. However, alterations in bone and/or gingival morphology (such as altered passive eruption or gingival recession) affecting the failing tooth and/or neighboring teeth to the extraction site could lead to incorrect planning. Sometimes, even the need for a new restoration in the adjacent teeth can lead to errors in planning. For these reasons, in all cases with extensive mucogingival deformities or buccal bone defects or when prosthetic correction in the sextant is needed, flap mobilization to achieve adequate management of hard and soft tissues must be considered [26].

Even when mucogingival enhancement is done, prosthetic modifications to achieve a proper esthetic outcome are needed. The management of the provisional’s emergence profile has been described in the literature to enhance the position and appearance of the soft-tissue contours [16,17,18,19].

This case report presents a multidisciplinary approach for replacing a failing tooth in the esthetic area with an immediate implant and the prosthetic rehabilitation of the adjacent teeth: the focus is on the prosthetic management of the provisional to achieve reconstruction of the peri-implant papillae.

## 2. Case Scenario

**Figure 1:**
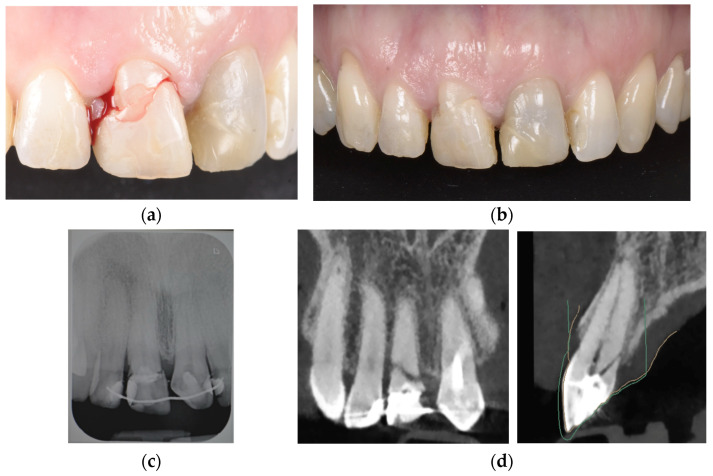
Baseline (January 2023). (**a**) Deep palatal crown-to-root fracture on the maxillary right incisor. The maxillary left incisor was necrotic and showed a fracture in the mesial portion of the crown; (**b**) the fractured fragment of 1.1 was temporarily bonded in place and splinted to adjacent teeth with an orthodontic ligature and flowable composite. Note that both 1.3 and 2.3 presented RT1 gingival; (**c**) periapical radiograph at the baseline; (**d**) CT section showing the deep root fracture. Comparative analysis of the soft tissue profile on CT (yellow line) and the planned profile from the preoperative diagnostic wax-up (green line).

A 52-year-old patient presented with a deep palatal crown-to-root fracture on the maxillary right central incisor. The maxillary left central incisor was necrotic and showed a mesial coronal fracture (Figure 1).

Based on physical and radiographic examination, the therapeutic options considered were:

- Orthodontic extrusion;
- Extraction of the maxillary right central incisor and IIP.

The patient was medically and periodontally stable without contraindications for surgery.

Due to the extension of the crown-root fracture and the patient’s neglect of orthodontic treatment, the tooth was deemed unrestorable. The fracture line extended significantly subgingivally, reaching beyond the interproximal bone crest; therefore, a crown lengthening procedure would have been highly invasive, with a considerable risk of compromising the adjacent teeth. Orthodontic treatment aimed at atraumatic extrusion had been planned but was not followed by the patient for aesthetic and time-related reasons. Hence, the treatment plan consisted of extraction and IIP.

### 2.1. Initial Prosthetic Phase

As an immediate resolution to the emergency consultation, the fractured fragment of the maxillary right central incisor was temporarily bonded in place and splinted to adjacent teeth with an orthodontic ligature and flowable composite. At the 1-month follow-up, the maxillary left central incisor tested negative for vitality and root canal therapy was performed; the tooth was reconstructed using a fiber post and composite.

Once the primary needs were solved, a prosthetic evaluation was carried out for the whole sextant.

**Figure 2:**
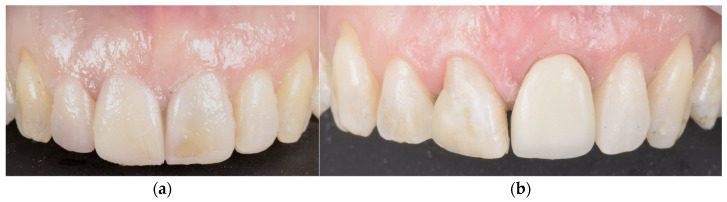
(**a**) Mock-up; (**b**) 1.2. and 2.2 restoration with direct composite; milled PMMA provisional on 2.1; the distal portion of the 1.1 was also prepared to create a wider diastema between 1.1 and 1.2 to enhance the papilla height.

Lateral incisors had marginal filtration on existing class IV as well as gingival recessions in both maxillary canines (RT1), which led to an inadequate pink evaluation score (PES) and white evaluation score (WES) [27,28]. Therefore, the treatment plan required a digital mock-up to study the golden proportion for centrals and laterals [29]. After patient approval, both lateral incisors were restored with direct composite restorations (Empress Direct Ivoclar). A vertical preparation of the maxillary left central incisor was done and a milled PMMA provisional restoration was relined and delivered [30]. A preparation of the contralateral central was also done and a diastema was left between both centrals for four weeks to allow space for the papilla and improve the vascular support for a future CTG [31] (Figure 2).

### 2.2. Pre-Surgical Evaluation

Before surgery, an intraoral scan was performed, and a Cone Beam Computer Tomography (CBCT) was requested to plan a fully guided implant insertion. The buccal bone was >1 mm and the amount of apical bone was sufficient to allow immediate placement and provisionalization of a 3.9 mm × 16 mm dental implant (V3, MIS Implants Technologies Ltd., Bar Lev Industrial Park, Israel). A 2 mm intermediate abutment was used to displace the implant-abutment connection away from the bone crest and reduce bone resorption (Connect abutment, MIS Implants Technologies Ltd., Bar Lev Industrial Park, Israel) and follow the one-abutment–one-time protocol [32]. The intermediate abutment’s cuff height was selected based on the prospective ideal position of the buccal soft-tissue margin of the final restoration. A virtual extraction of the maxillary right central incisor and the ideal three-dimensional placement of the implant were performed in a virtual planning software (MSOFT Planning System v2.16). In an apical-coronal direction, the neck of the endosseous implant portion was positioned 3.5–4 mm apical of the ideal zenith point and engaging the bone beyond the apex of the extraction socket (4–5 mm deep) near the palatal wall to achieve proper primary stability. Sagittally, the implant was planned to be placed palatally, with a minimum of 1.5 mm of gap between the implant and the facial bony wall of the extraction socket, and a 1 mm gap from the palatal bone: an angled screwed abutment was chosen to allow for a screw-retained prosthesis.

### 2.3. Surgical Procedure

**Figure 3:**
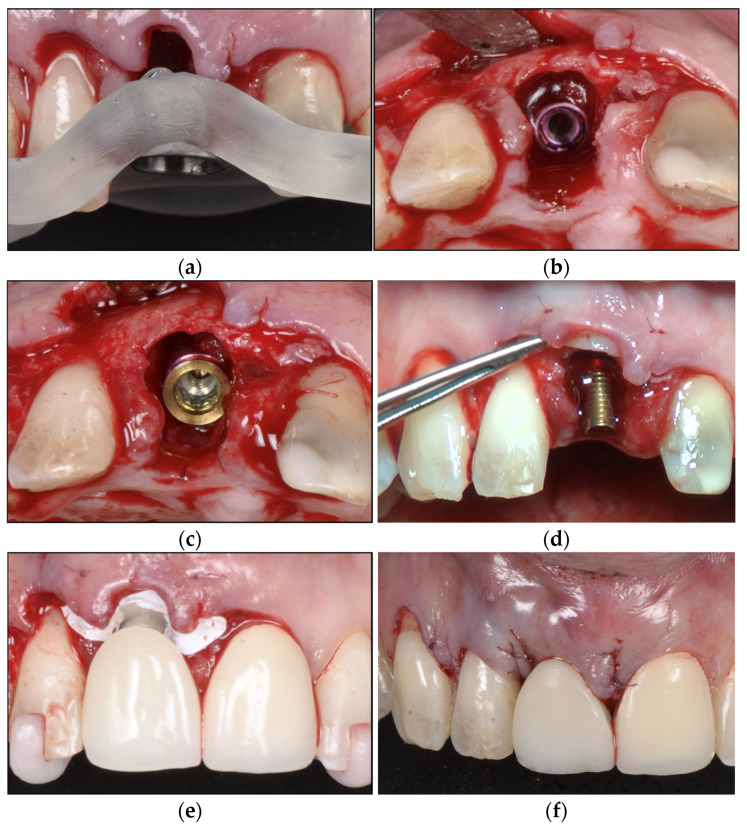
Surgical phase (April 2023). (**a**) Surgical guided implant positioning. (**b**) Implant positioned. (**c**) Connect Abutment screwed. (**d**) Connective Tissue Graft sutured to the inner aspect of the buccal flap. (**e**) Immediate provisional crown during the relining phase. (**f**) Flap suture.

The surgery was performed by an expert clinician (Prof. Martina Stefanini) (Figure 3). A coronally advanced envelope flap with a frontal approach was performed according to the mucogingival approach previously described by Stefanini et al. [11], using a split–full–split dissection to allow tension-free coronal advancement and simultaneous management of the hard and soft tissues.

Anatomic papillae were carefully preserved to guarantee the future adaptation and stability of the flap at the time of suturing. Following flap elevation, the right central incisor was atraumatically extracted while preserving the buccal bone plate and the adjacent anatomical papillae. A fully guided immediate implant placement protocol was then performed according to the preoperative digital planning. The implant (V3, MIS Implants Technologies Ltd., Bar Lev Industrial Park, Israel) achieved primary stability greater than 35 Ncm, allowing immediate provisionalization. The peri-implant gap was grafted with a xenogeneic bone substitute (cerabone^®^, Botiss biomaterials GmbH, Berlin, Germany) [5,33] and a connective tissue graft (CTG) [34] harvested from the posterior palate was positioned on the inner aspect of the buccal flap to enhance peri-implant soft tissue thickness and stability. The 2 mm intermediate abutment was screwed onto the fixture (25 N). The provisional restoration had been fabricated before surgery based on the digital implant planning and was relined chairside after implant placement. Particular attention was devoted to creating an initially under-contoured and polished emergence profile in order to provide space for soft tissue maturation during healing. This prosthetic design represented the starting point for the subsequent soft tissue conditioning phase that ultimately led to the development of the B.U.M.P. concept.

The temporary crown was then delivered at 25 N.

The flap was coronally advanced and stabilized with sling sutures to achieve passive closure and intimate adaptation of the tissues around the provisional restoration. After surgery, the soft tissues appeared stable, with no evidence of tension or blood seepage from the wound margins. Postoperative instructions included chlorhexidine rinses (0.12%) twice daily for two weeks and avoidance of mechanical plaque control in the surgical area during the initial healing phase.

### 2.4. Post-Surgical Prosthetic Conditioning and Finalization

The immediate provisional in the anterior area requires accurate control of static and dynamic occlusal interferences to avoid complications during osteointegration. Moreover, attention was paid to drawing a non-compressive emergence profile [35], to allow the soft tissues to rebound without any impingement throughout the healing period. At 6 months, implant integration was confirmed with radiographs and clinical controls, but the soft tissue presented shrinkage of the distal papilla, which negatively influenced the PES [36]. Prosthetic conditioning of the buccal and interproximal profiles of the provisional was needed to improve the esthetic appearance of the restoration.

A modification of the buccal aspect was done by reducing the internal surface of the gingival contour with a diamond bur, and by adding new composite material to the Crestal (C) and Bounded (B) zones [16].

This modification with a steeper profile was left in place for 1 month to sustain buccal scalloping at a more apical level, to emulate the contralateral tooth, and promote coronal papilla displacement in the distal interproximal space. However, after a 2-month observation period, the growth was not clinically significant; thus, a second modification at the Transition zone of the emergence profile was performed [37].

**Figure 4:**
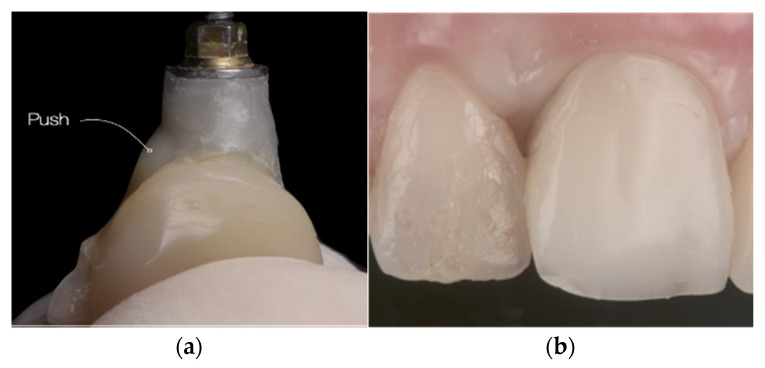
Provisional modification (December 2023). (**a**) Composite bump to push the distal interproximal soft tissues; (**b**) Conditioning of the distal papilla by the composite Biologic Under-proximal Morphology Profile: please note the ischemia of the tissues at the time of provisional placement.

This modification implied the creation of a composite “bump” (Figure 4), which consists of an asymmetrical design of the temporary restoration under the proximal area to generate a targeted push of the tissues and the papilla from palatal to coronal [7] (hence, Biologic Under-proximal Morphology Profile B.U.M.P.).

This unconventional design of the emergence profile, together with the ongoing soft-tissue rebound [38], helped develop a narrower, more coronal interproximal soft tissue, which improved the overall PES of the case.

**Figure 5:**
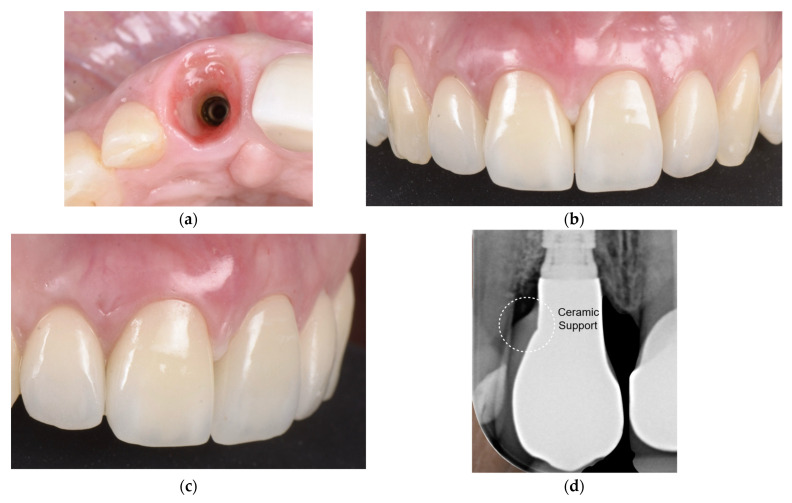
Finalization (April 2024). (**a**) Before final digital impression: note the soft tissue thickness in the buccal aspect of the right maxillary incisor and the area where the composite “bump” compresses the under-proximal part of the distal papilla; (**b**,**c**) Frontal view of the final restorations of the second sextant. (**c**) Detail of the distal papilla of the final crown on implant 1.1. (**d**) Radiographic view of the final crown on implant 1.1: the ceramic support in the under-proximal part of the distal papilla is visible, which has been recreated identically to that of the temporary crown.

Once the desired position of the tissues was achieved and stabilized, guided by the “bump” prosthetic design, the case could be finalized with definitive restorations (Figure 5).

In such a singular situation, it is mandatory to copy the atypical emergence profile obtained by the customization with a composite “bump” from the provisional to the final restoration, maintaining the papilla support.

Digital impressions using the RepliCAD approach were done to allow a precise copy from the provisional to the final restoration [39]. The final restorations included a veneered zirconia crown on implant 1.1, a veneered zirconia crown on tooth 2.1 and two feldspathic veneers on 1.2 and 2.2.

The crown on 1.1 was directly screwed in, and the crown on 2.1 was cemented following the SAL approach [40] using a resin cement. The use of the “Bump” approach resulted in an optimal esthetic outcome for the interproximal tissues.

**Figure 6:**
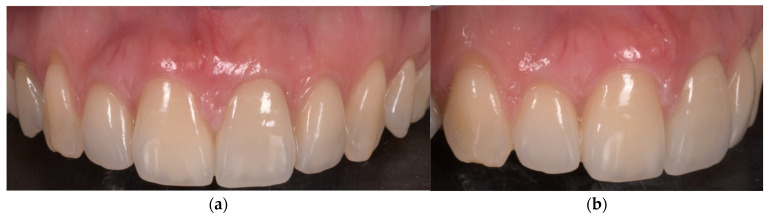
Twelve-month follow-up (April 2025). (**a**) Frontal view. (**b**) Lateral view.

The 12-month follow-up pictures (Figure 6) show perfect tissue stability and excellent soft tissue aesthetics.

## 3. Discussion

IIP was traditionally associated with a higher risk of buccal soft tissue dehiscence [41] and esthetic compromise [21,42]. A recent systematic review noted that the risk of this unpleasant event can be minimized with the adjunctive use of CTG and the filling of the bone-to-implant gap with biomaterials [5]. A 2023 review by Stefanini et al. [43] found that sites with soft tissue augmentation demonstrated greater long-term stability in both peri-implant soft tissue volume and marginal bone levels. In contrast, implants that did not receive augmentation were sometimes subject to downward movement of the gingival margin over time. This is due to the CTG’s ability not only to remain stable over time but also to progressively mature and increase in size through creeping, thereby determining excellent results with regard not only to general aesthetics and peri-implant health but also to the appearance of the interproximal soft tissues.

Tavelli et al. [44] identified a mucosal thickness (MT) of 2.23 mm, measured 3 mm apical to the mucosal margin, as a critical threshold. When at least 2.23 mm of MT and a stable, adherent keratinized mucosa (KM) band were present, the soft tissue margin remained stable for five years, irrespective of buccal bone position or dimension.

This clinical case illustrates that a combined surgical and prosthetic protocol can achieve optimal functional and esthetic outcomes following immediate implant placement. A maxillary central incisor that was to be extracted was successfully substituted with an immediate implant, due to the adjunctive application of a CTG and immediate provisionalization. A full-flap approach was intentionally selected over a flapless technique to ensure precise control of the surgical field. The mucogingival design, performed using a split-full-split dissection without vertical releasing incisions, preserved the buccal bone integrity, facilitating tooth extraction and immediate implant placement in a prosthetically driven position.

CTG is a minimally invasive surgical procedure that ensures the stability of the flap in the immediate post-surgical period. As the CTG matures and thickens during the provisionalization, it helps peri-implant papillae grow in height and prevents future peri-implant dehiscence. The mucogingival technique, characterized by partial-thickness incision of the surgical papillae toward the target site, provided both stabilization during suturing and tension-free coronal advancement, thus reducing the likelihood of postoperative contraction and enhancing esthetic stability.

Digital planning and computer-guided surgery were instrumental in achieving precise three-dimensional implant positioning. Preoperative virtual planning enabled the fabrication of an immediate provisional restoration before the surgical procedure, optimizing primary stability and reducing intraoperative time and patient morbidity.

These concepts are consistent with the protocol proposed by Gamborena et al. [45], who described a comprehensive immediate implant workflow based on digital planning, customized provisional restorations, connective tissue grafting, and prosthetically guided conditioning of the peri-implant soft tissues. Their approach highlights the importance of the emergence profile as a key determinant of soft tissue maturation and long-term esthetic stability.

Located between the implant platform and the soft-tissue cervical margin, the emergence profile is responsible for the transition from the implant’s cylindrical shape to the final contour of the crown. In this clinical case, the use of an intermediate abutment together with the concept of one-abutment one-time helped to minimize the trauma at the implant-abutment interface during the multiple disconnections that occurred [46]. The progressive modification of the temporary restoration with the composite was decisive in promoting the conditioning and maturation of the peri-implant tissues previously thickened with the application of the CTG.

**Figure 7:**
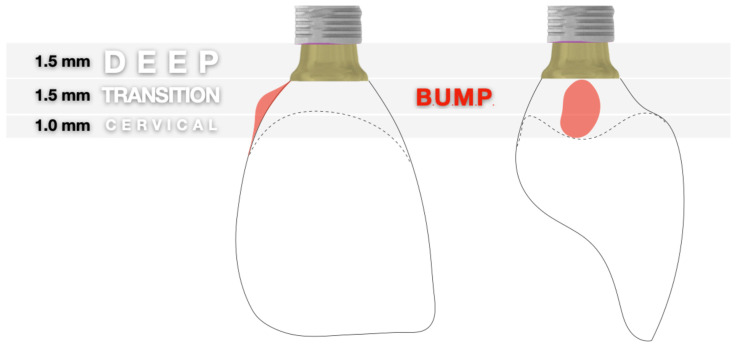
Schematic representation of the Biologic Under-proximal Morphology Profile (B.U.M.P.) concept.

A key innovation in this case was the implementation of the Biologic Under-proximal Morphology Profile (B.U.M.P.) concept (Figure 7). The customized emergence profile, introduced during the secondary modification of the provisional, was designed to guide soft tissue modeling coronally and vestibulary. The strategic use of composite protrusions in the interproximal zone provided structural support for soft tissue remodeling, resulting in complete closure of the interdental embrasure and the elimination of the black triangle, thereby improving the PES.

Finally, the accurate transfer of the subgingival and supragingival morphology from the provisional to the definitive restoration was essential to maintaining the achieved soft-tissue support. The duplication of the customized contours ensured that the peri-implant papillae and marginal soft tissues remained stable between provisionalization and final prosthesis delivery, underpinning the long-term esthetic and biological success of the rehabilitation.

Recently, biologically oriented treatment concepts have been proposed to optimize peri-implant tissue stability. For example, Grenzi et al. [47] introduced the Subperiosteal Peri-Implant Augmented Layer (SPAL) technique to improve peri-implant hard tissue support in IIP through a minimally invasive regenerative approach. Although primarily focused on hard tissue augmentation, this concept shares with the B.U.M.P. technique the objective of enhancing peri-implant tissue stability by combining biologically driven surgical principles with careful prosthetic planning. Unlike the SPAL technique, however, the B.U.M.P. concept specifically targets papilla maturation through progressive modification of the provisional restoration.

The B.U.M.P. approach may be indicated in single implant rehabilitations in the aesthetic zone presenting peri-implant soft tissue deficiencies, loss or flattening of the interproximal papilla, reduced soft tissue volume, or compromised emergence profile requiring enhanced mucosal contouring and tissue support. The technique may be particularly useful in patients with high aesthetic demands and favorable local anatomical conditions, including adequate bone support and sufficient soft tissue quality. Conversely, the procedure should be considered with caution or avoided in patients presenting poor plaque control, untreated or unstable periodontal disease, heavy smoking habits, limited soft tissue availability, compromised vascularization, or systemic conditions potentially affecting wound healing and long-term tissue stability. Careful patient selection and maintenance are therefore essential for achieving predictable aesthetic outcomes.

**Figure 8:**
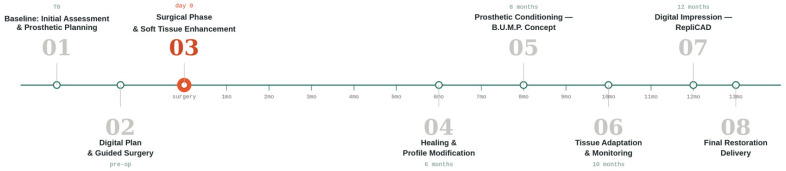
Clinical flowchart and timeline of the Biologic Under-proximal Morphology Profile (B.U.M.P.) concept.

Figure 8 schematically represents the clinical flowchart of the Biologic Under-proximal Morphology Profile (B.U.M.P.) concept.

This report represents an initial proof of concept and therefore does not allow conclusions regarding the predictability or generalizability of the proposed protocol. Patient-related factors, operator experience, and anatomical variability may influence the outcome. Additional larger case series or controlled clinical studies and long-term follow-up are required to validate the reproducibility and clinical benefits of this protocol.

## 4. Conclusions

IIP with immediate provisionalization is the best option in the aesthetic area, for both clinicians and patients. Unfortunately, it is not suitable for every case, which is why it is mandatory to perform an accurate evaluation before surgery. The absence of a quantity of bone apical to the element to be extracted, the possibility of performing an atraumatic extraction, and, above all, the absence of an untreated periodontal pathology are all factors that can compromise the possibility of this type of treatment.

From a prosthetic perspective, it is important to integrate biological, surgical, and prosthetic concepts to achieve optimal restoration integration. Periodontal plastic surgery can prepare soft tissues for prosthetic conditioning and optimize the camouflage of implant reconstruction. Improper management of prosthetic contours during the various phases of treatment can undermine even the best surgical treatment. For this reason, the choice of the right implant-prosthetic components and the proper emergence profiles is of paramount importance for achieving and maintaining the result over time. Digital workflows (from surgical planning to the final impression) are another key factor in achieving predictable, accurate clinical outcomes.

Within the limitations of this case report, this clinical scenario illustrates how this novel combined surgical and prosthetic approach, preceded by proper soft-tissue surgical management and augmentation, with the Biologic Under-proximal Morphology Profile that accompanies soft tissue creeping, can be crucial for achieving the ideal esthetic in implant-supported rehabilitation.

Controlled randomized clinical trials with long follow-up are required to confirm the efficacy of this concept.

## Data Availability

The original contributions presented in this study are included in the article. Further inquiries can be directed to the corresponding author.

## Abbreviations

The following abbreviations are used in this manuscript:

IIP	Immediate Implant Placement
CTG	Connective Tissue Graft
PES	Pink Evaluation Score
WES	White Evaluation Score
BUMP	Biological Under-proximal Morphology Profile
